# Development of a nanocopper-decorated laser-scribed sensor for organophosphorus pesticide monitoring in aqueous samples

**DOI:** 10.1007/s00604-022-05355-w

**Published:** 2022-06-13

**Authors:** David Bahamon-Pinzon, Geisianny Moreira, Sherine Obare, Diana Vanegas

**Affiliations:** 1 Department of Environmental Engineering and Earth Sciences, Clemson University, Clemson, SC USA; 2 Global Alliance for Rapid Diagnostics, Michigan State University, East Lancing, MI USA; 3 Joint School of Nanoscience and Nanoengineering, North Carolina A&T State University and UNC Greensboro, Greensboro, NC USA; 4 Interdisciplinary Group for Biotechnology Innovation and Ecosocial Change -BioNovo, Universidad del Valle, Cali, Colombia

**Keywords:** Organophosphorus pesticides, Glyphosate, LIG, Sensor, Amperometry, Turbostratic graphene, Copper nanoparticles, Environmental monitoring

## Abstract

Organophosphorus pesticides are widely used in industrial agriculture and have been associated with water pollution and negative impacts on local ecosystems and communities. There is a need for testing technologies to detect the presence of pesticide residues in water sources, especially in developing countries where access to standard laboratory methods is cost prohibitive. Herein, we outline the development of a facile electrochemical sensor for amperometric determination of organophosphorus pesticides in environmental water samples. A three-electrode system was fabricated via UV laser-inscribing on a polyimide film. The working electrode was functionalized with copper nanoparticles with affinity toward organophosphate compounds. The sensor showed a limit of detection (LOD) of 3.42 ± 1.69 µM for glyphosate, 7.28 ± 1.20 µM for glufosinate, and 17.78 ± 7.68 µM for aminomethylphosphonic acid (AMPA). Sensitivity was highest for glyphosate (145.52 ± 36.73 nA⋅µM^−1^⋅cm^−2^) followed by glufosinate (56.98 ± 10.87 nA⋅µM^−1^⋅cm^−2^), and AMPA (30.92 ± 8.51 nA⋅µM^−1^⋅cm^−2^). The response of the sensor is not significantly affected by the presence of several ions and organic molecules commonly present in natural water samples. The developed sensor shows promising potential for facilitating environmental monitoring of organophosphorus pesticide residues, which is a current need in several parts of the world.

**Graphical Abstract:**

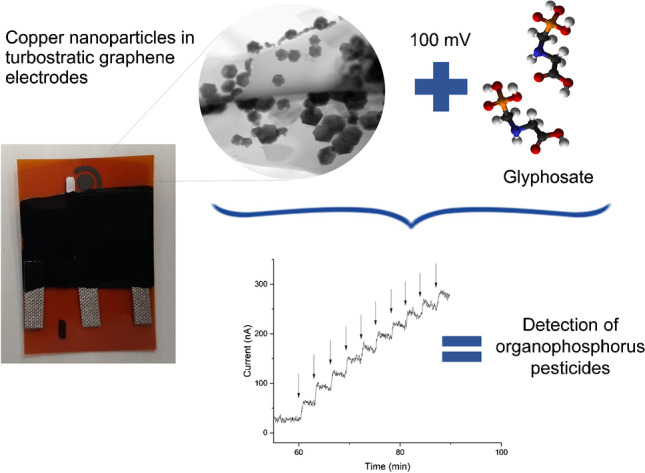

**Supplementary Information:**

The online version contains supplementary material available at 10.1007/s00604-022-05355-w.

## Introduction

Organophosphorus pesticides have been extensively used in industrial agriculture. For instance, glyphosate [*N*-(phosphonomethyl)glycine] is the most used herbicide in the world [1], with applications in monocultures of genetically modified crops [2]. However, glyphosate can potentially pollute soil, water bodies, and crops and negatively affect non-targeted organisms [3]. Moreover, glyphosate has been found in human urine samples [4], and it has been associated with endocrine system disruption and DNA damage in humans [3]. In 2015, the World Health Organization (WHO) and the International Agency in Research on Cancer (IARC) classified glyphosate in Group 2A as *probably carcinogenic to humans* [5]. The human health impacts from exposure to glyphosate and its primary residue, aminomethylphosphonic acid (AMPA), is an ongoing debate in the scientific community. Similarly, regulatory frameworks for glyphosate use vary vastly depending on the country. For instance, glyphosate has a maximum residue limit (MRL) in drinking water of 700 μg⋅L^−1^ (4.14 μM) in the USA, according to the EPA [6] and 0.1 μg⋅L^−1^ (0.59 nM) in the European Union [7, 8].

In this context, it is necessary to facilitate environmental monitoring of glyphosate with a high spatial and temporal resolution, which is a critical need in parts of the world where standard laboratory techniques are inaccessible or cost-prohibitive. Currently, there are several technologies for pesticides detection in laboratory settings, including liquid chromatography-mass spectrometry (LC/MS) and high-performance liquid chromatography (HPLC). However, these methodologies generally require trained personnel, do not allow in situ and real-time monitoring, and are expensive and time consuming [9]. Other techniques, such as enzyme-linked immunosorbent assay (ELISA), use antibodies, which can be expensive to produce and may require low-temperature storage [10].

Considering the limitations of conventional laboratory-based techniques for applications in developing countries, it is necessary to develop detection methodologies that are affordable and allow for rapid and in situ assessment of water pollutants. Portable sensors operate based on molecular interactions occurring at the sensor/sample interface. These analytical devices transform a molecular recognition event into a measurable signal, using an electrochemical or optical transduction mechanism connected to a signal acquisition system [11].

To date, few electrochemical sensors for glyphosate detection have been reported in the scientific literature. For example, Do et al. (2015) developed a sensor based on molecularly imprinted polymers functionalized with gold nanoparticles for linear sweep voltammetry detection of glyphosate residues in soybeans [9]. Moraes et al. (2010) used multi-walled carbon nanotubes with copper phthalocyanine in a glassy carbon electrode for differential pulse voltammetry detection of pesticides in sodium phosphate buffer [12]. Cao et al. (2019) developed a metal–organic framework platform based on copper and 1,3,5-benzenetricarboxylic acid (BTC) immobilized on a tin oxide electrode for differential striping pulse voltammetry quantification in soybeans [13]. Poorahong et al. (2015) demonstrated the development of an electrochemical sensor based on a gold electrode electrodeposited with copper nanowires for amperometric testing of fresh fruit and vegetable samples [14]. Even though previously reported sensors showed high-performance capabilities, the type of materials and sophisticated fabrication techniques make these technologies hardly reproducible in resource-constrained regions. Moreover, the need for hazardous reagents in some these tests discourages the use of the technology outside of laboratory settings [15].

Turbostratic graphene obtained from laser engraving onto polyamide substrates has emerged as a relatively low-cost material suitable for developing sensing platforms [16]. Herein, we report on the artisanal manufacture of a portable sensor for assessment of organophosphorus pesticides residues in environmental water samples. The working mechanism of the sensor is based on the anodic current response generated from the formation of a complex between the nanocopper on the sensor surface and the functional groups in the organophosphate pesticides [14, 17]. The test is performed in an electrochemical cell maintained at neutral pH and low overpotential without the use of hazardous chemicals.

## Experimental section

### Materials and reagents

Copper sulfate (CuSO_4_), ethanol (C_2_H_5_OH), potassium ferrocyanide (K_4_Fe(CN)_6_), phosphate-buffered saline (PBS) solution, sodium sulfate (Na_2_SO_4_), magnesium chloride (MgCl_2_), aluminum chloride (AlCl_3_), mercury (II) nitrate hydrate (H_2_HgN_2_O_7_), sodium hydroxide (NaOH), hydrochloric acid (HCl), ammonium nitrate (NH_4_NO_3_), humic acid salt, atrazine, glufosinate-ammonium, and chlorpyrifos were obtained from Fisher Scientific (Waltham, MA, USA). Potassium nitrate (KNO_3_), potassium chloride (KCl), sodium bicarbonate (NaHCO_3_), calcium chloride (CaCl_2_), magnesium sulfate (MgSO_4_), ammonium chloride (NH_4_Cl) silver/silver chloride paste (Ag/Ag/Cl), glyphosate [N-(phosphonomethyl)glycine], (aminomethyl)phosphonic acid (AMPA), phosphoric acid (H_3_PO_4_), and a certified reference material 44,690-U for glyphosate (1000 μg⋅mL^−1^ solution in distilled water) were purchased from Sigma Aldrich Inc. (St. Louis, MO, USA). Kapton™ (polyimide) film (electrical grade polyimide film, 0.0050″ thick) was obtained from McMaster-Carr (Elmhurst, IL, USA). Calcium sulfate was obtained from Watson (Caruthers, California, USA). Roundup® was bought from a local agricultural store (Bayer Inc., Whippany, NJ, USA).

### Sensing platform fabrication

Laser-inscribed graphene electrodes (LIG) were fabricated based on the methodology by Tehrani and Bavarian (2016) [18]. A three-electrodes system (working, reference, and counter electrodes) was designed and scribed on Kapton film using a UV laser engraver (NEJE Laser Engraver Printer, 1500mW, 490 × 490 Pixel) to obtain laser-inscribed graphene (LIG) electrodes. The following parameters were used during laser scribing: distance from the lens to the surface of the sample: 8 cm, number of scans: 2, burning time: 15 ms, brightness: 30%. Silver chloride paste (Ag/AgCl) was applied to the reference electrode, and a metallic tape was incorporated at the terminal of each electrode to protect the LIG from abrasion damage during the connection of the bonding pads with the potentiostat. A layer of nitrocellulose lacquer was applied as passivation material on the surface of the electrodes’ stems.

Copper nanoparticles were incorporated on the working electrode via electrodeposition in a solution of 250 mM copper sulfate (CuSO_4_) and 2.5 mM sodium sulfate (Na_2_SO_4_), according to the process described by Vanegas et al. [16]. A copper rod was used as the anode and the working electrode as the cathode. A constant potential of 9 V was applied for 1 s. Prior to the electrodeposition step, the copper rod was electropolished with a solution consisting of 25% ethanol and 25% phosphoric acid for 30 s at 9 V to remove any impurities from its surface. A scheme of the fabrication process of the electrodes is shown in Fig. S1. Electrodes with similar electrochemical response were selected using cyclic voltammetry (Fig. S2).

### Material characterization

Scanning electron microscopy (SEM) and energy-dispersive X-ray spectroscopy (EDS) were performed in a scanning electron microscope SU5000 with an accelerating voltage of 5 kV to study the morphology and elemental composition of LIG and LIG-Cu electrodes. X-ray diffraction (XRD) analysis was performed in a PANalytical Empyrean at a potential of 45 kV and a current of 40 mA. X-ray photoelectron spectroscopy (XPS) analysis of LIG-Cu was conducted in an XPS/UVS-SPECS System using a PHOIBOS 150 Analyzer with an anode voltage of 10 kV, power of 300 W, and emission current of 30 mA.

### Electrochemical performance characterization

DC-potential amperometry (DCPA) was selected as the detection technique for glyphosate testing. DCPA experiments were performed using a portable potentiostat (ABE-Stat, Diagenetix, Honolulu, USA [19]). The following settings were applied: constant potential of 100 mV, polarization time of 60 min, and continuous stirring at 450 rpm. 20 mL of PBS buffer (pH: 7.2) was used as the working solution for DCPA testing. To generate a calibration curve, 10 µL aliquots of glyphosate solution (8 mM) were successively injected into the electrochemical cell every 3 min. Each glyphosate addition generated a change in electrical current, which was recorded using the ABE STAT software (Diagenetix, Honolulu, USA).

Performance parameters were calculated based on the DCPA curves. The analytical sensitivity is equal to the slope of the linear portion of the calibration curve (Eq. 1) divided by the geometric surface area of the working electrode (0.05 cm^2^).

1 $${i=sC+i}_{0}$$

where:

*i*: current (nA)
*s*: slope (nA⋅µM^−1^)
*i*_o_: current intercept (nA)

The lower limit of detection (LOD) was calculated as:

2 $$LOD=3\sigma /s$$

Where:

*σ*: standard deviation of the baseline (nA)
*s*: slope of the calibration curve (nA⋅µM^−1^)

Finally, the response time (*t*_95_) was obtained by fitting the data from three successive steps changes in concentration to the exponential rise to a maximum model (Eq. 4), using the sum of chi-square to minimize the error. The response time is defined as the time when 95% of the stable response is obtained (Eq. 4) [18]. The performance parameters of the sensor were also calculated using the commercial formulation Roundup, AMPA, and other organophosphorus compounds (glufosinate-ammonium and chlorpyrifos) as analytes.

3 $${i}_{95}={i}_{0}+a\left(1-{e}^{-{\mathrm{bt}}_{95}}\right)$$

where:

*i*_*95*_: current obtained at the response time (µA)
*i*_*o*_: baseline of each step (µA)
*a*: upper limit or stable response (µA)
*b*: model constant
*t*_*95*_: response time (s)

4 $${t}_{95}=\frac{-\mathrm{ln}(0.05\left(\frac{{i}_{0}}{a}+1\right))}{b}$$

The effect of the pH, selectivity, and stability of the sensor was evaluated using an amperometric test. For all the experiments, the electrodes were polarized at 100 mV in 20 mL of PBS buffer (pH 7.2) during one hour. The detailed methodology for each analysis, including the determination of the electroactive surface area (ESA), can be found in the supplemental information.

### Statistical analysis

ANOVA was used to determine significant differences amongst three or more treatments (*p* < 0.05). A Tukey test was performed for pair-wise comparison whenever treatments were significantly different. For 2-treatments comparison, a *t*-test (*p* < 0.05) was performed. Table S1 shows the test, levels, and response variables for each analysis. The software JMP (JMP Pro 16, SAS Institute Inc., Cary, NC, USA) was used for all statistical analyses.

## Results and discussion

### Materials characterization

**Fig. 1:**
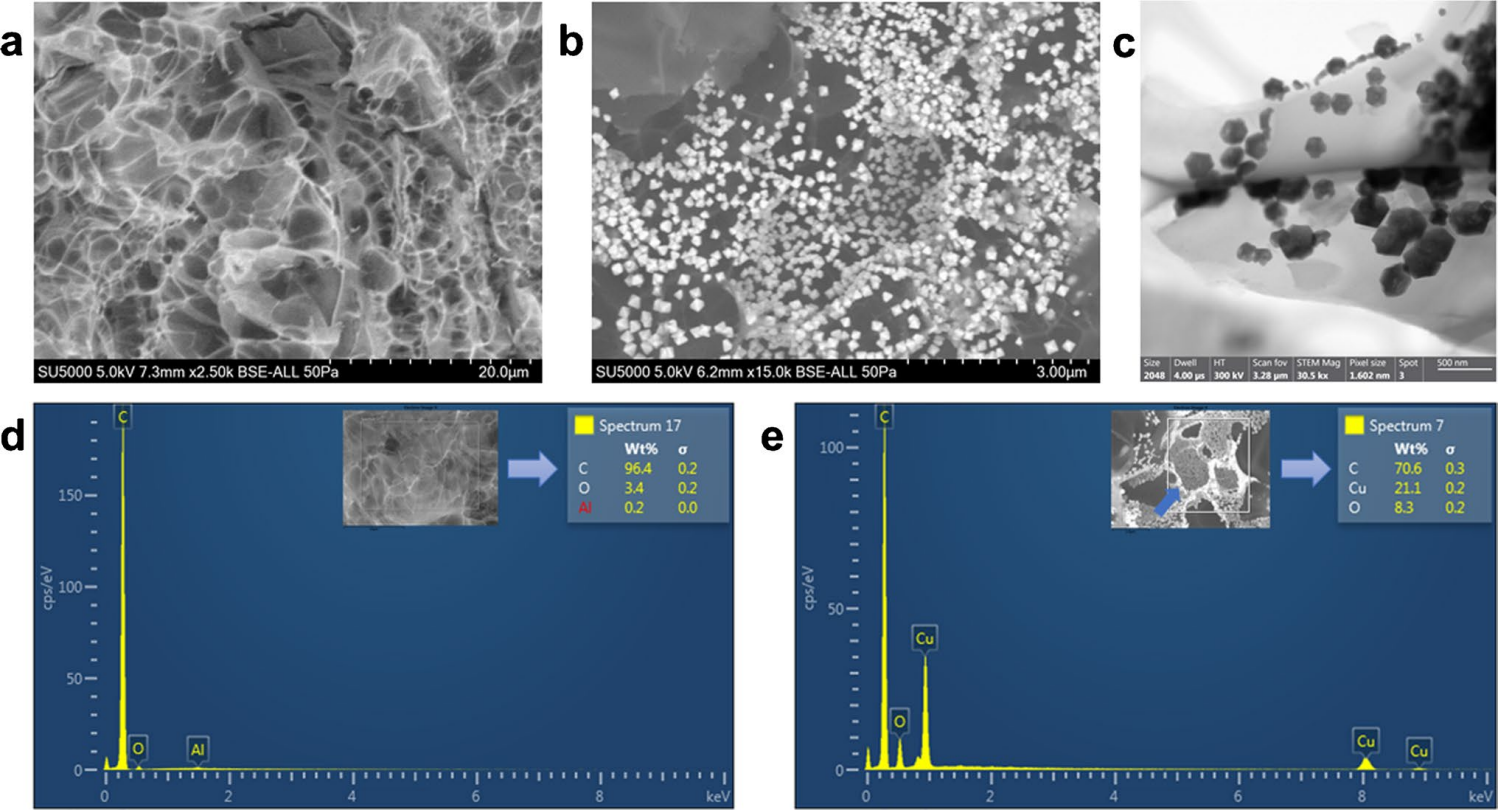
Scanning Electron Microscopy (SEM) images of **a** laser-inscribed graphene (LIG) with a magnification of 2.50 k and **b** LIG with copper nanoparticles (LIG-Cu) with a magnification of 10.0 k. **c** Scanning transmission electron microscopy (STEM) of LIG-Cu electrodes. Energy-dispersive X-ray spectroscopy (EDS) curves of **d** LIG and **e** LIG-Cu

Laser-inscribed graphene electrodes (LIG) and LIG modified with copper nanoparticles (LIG-Cu) were characterized in terms of morphology and elemental composition using scanning electron microscopy (SEM), scanning transmission electron microscopy (STEM), energy-dispersive X-ray spectroscopy (EDS), and X-ray diffraction (XRD). Figure 1a shows the SEM micrographs of the LIG surface, with a sponge-like morphology, similar to the SEM images obtained by Mogera et al. (2015) for carbonaceous materials based on laser-assisted transformation of biomass materials [20]. Unlike Bernal stacked graphitic carbon nanomaterials, the LIG electrode surface displays random rotation between adjacent carbon layers, as well as misaligned stacking, which are characteristic features of highly decoupled turbostratic graphene materials [20, 21]. According to the EDS analysis (Fig. 1d), the LIG electrode surface is composed of 96.4% of carbon and 3.4% of oxygen. Figure 1b shows the SEM micrograph of the LIG electrode after copper electrodeposition (i.e., LIG-Cu). As seen in the Fig. 1b and S3a, copper nanoparticles were successfully synthesized on the surface of the LIG electrodes, resulting in the relatively homogeneous distribution of crystal-shaped nanoparticles with a size ranging from 80 to 500 nm (Fig. 1c and S3b-c). According to Huang et al. (2005), electrocrystallization of copper onto carbon electrodes is tightly dependent on nucleation overpotential and time [22]. In this case, we presume that the process of copper electrodeposition onto LIG electrodes follows progressive nucleation kinetics with three-dimensional diffusion-controlled growth. Tehrani and Bavarian (2016) obtained similar results with copper nanocubes electrodeposited on the surface of graphene electrodes [17]. The EDS spectrum of LIG-Cu (Fig. 1e) reveals several peaks for copper and a strong peak for oxygen that does not appear in the LIG spectrum. The resulting surface composition of LIG-Cu is 70.6% of carbon, 21.1% of copper, and 8.3% of oxygen (Fig. 1d, e). The increasing oxygen content on the surface of the electrode can be explained by the appearance of copper oxide, which was confirmed via XPS characterization (Fig. S4a). The XRD spectrum for LIG-Cu electrodes (Fig. S4b) shows four peaks at 2θ of 36.4, 42.3, 61.4, and 73.5°, confirming the presence of copper oxide (Cu_2_O) in the surface of the electrodes.

### Glyphosate detection

**Fig. 2:**
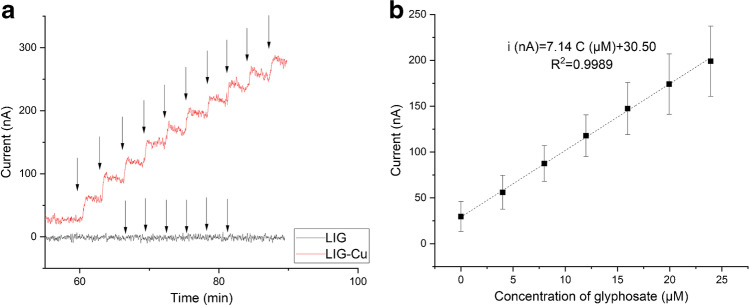
The **a** Representative amperometric response of LIG and LIG-Cu electrodes in PBS solution (pH 7.2) at a polarization potential of 100 mV (rolling average, *n* = 5). Black arrows represent injections of a glyphosate solution to the electrochemical cell. **b** Calibration curve of LIG-Cu electrodes in the presence of glyphosate (error bars represent standard deviation; *n* = 18)

Amperometry was used to examine the response of the sensor to the presence of glyphosate within the 4–24 µM concentration range. We applied a constant potential of 100 mV during 60 min as a conditioning process that results in a stable current response [16]. Figure 2a shows a representative real-time DCPA experiment in which LIG and LIG-Cu working electrodes were exposed to increasing concentrations of glyphosate. As seen in this figure, the current response of bare LIG electrodes was not significantly affected by the exposure to the analyte (Fig. 2a, black curve). On the other hand, a typical staircase amperometric response was obtained after the injection of glyphosate to the electrochemical cell using LIG-Cu electrodes (Fig. 2a, red curve). Additionally, the ESA of LIG-Cu (0.035 ± 0.004 cm^2^) was higher than LIG (0.026 ± 0.002 cm^2^), indicating a higher conductivity of the LIG-Cu electrodes (Fig. S5). The change of oxidative current in the presence of glyphosate is mediated through three mechanisms: (i) the deprotonation of the glyphosate molecule to form divalent cations at neutral pH, (ii) the formation of copper and copper oxide in the surface of the electrode when a potential of 100 mV is applied at a pH of 7.2 [23], and (iii) the complexation between deprotonated glyphosate and copper at a pH of 7.2 in aqueous solution [17], which results in an anodic current.

Thus, the oxidative current at each step is correlated with the concentration of glyphosate in the electrochemical cell, with strong linearity within the concentration range tested in the experiments (*R*^2^⪫ 0.99) (Fig. 2b). The limit of detection (LOD), sensitivity, and response time of the sensor are 3.42 ± 1.69 µM, 145.52 ± 36.73 nA⋅µM^−1^⋅cm^−2^, and 62.00 ± 13.02 s, respectively. The average LOD of the sensor is slightly lower than the MRL established by the EPA (700 μg⋅L^−1^ or 4.14 µM), which demonstrates its usability for in-field screening of glyphosate pollution. When the LIG-Cu electrode is connected to a high-end benchtop potentiostat (MultiPalmSens4, PalmSens) instead of a low-cost portable potentiostat (ABE-Stat), the LOD was as low as 0.69 ± 0.32 µM, and the sensitivity was 111.76 ± 13.44 nA⋅µM^−1^⋅cm^−2^ (Fig. S6), which demonstrates the potential applicability of the sensor as an analytical laboratory tool. This is an attractive option for regions with limited laboratory capacity since the cost of the standard equipment, supplies (including columns), and reagents for glyphosate testing (e.g., HPLC-FLD, LC–MS/MS) is considerably higher than the cost of a benchtop potentiostat. Let alone the intensive training of personnel, equipment maintenance, building infrastructure (including ambient controls), and other sources of increased cost that can be avoided by the relative simplicity of electrochemical testing.

The effect of the pH on the performance of the LIG-Cu sensor was evaluated. As observed in Fig. S7, the highest amperometric response from a single injection of analyte is obtained within the pH range from 7 to 8. Thus, we selected PBS (pH 7.2) as the buffer for the detection tests.

### Effect of different organophosphorus pesticides

**Fig. 3:**
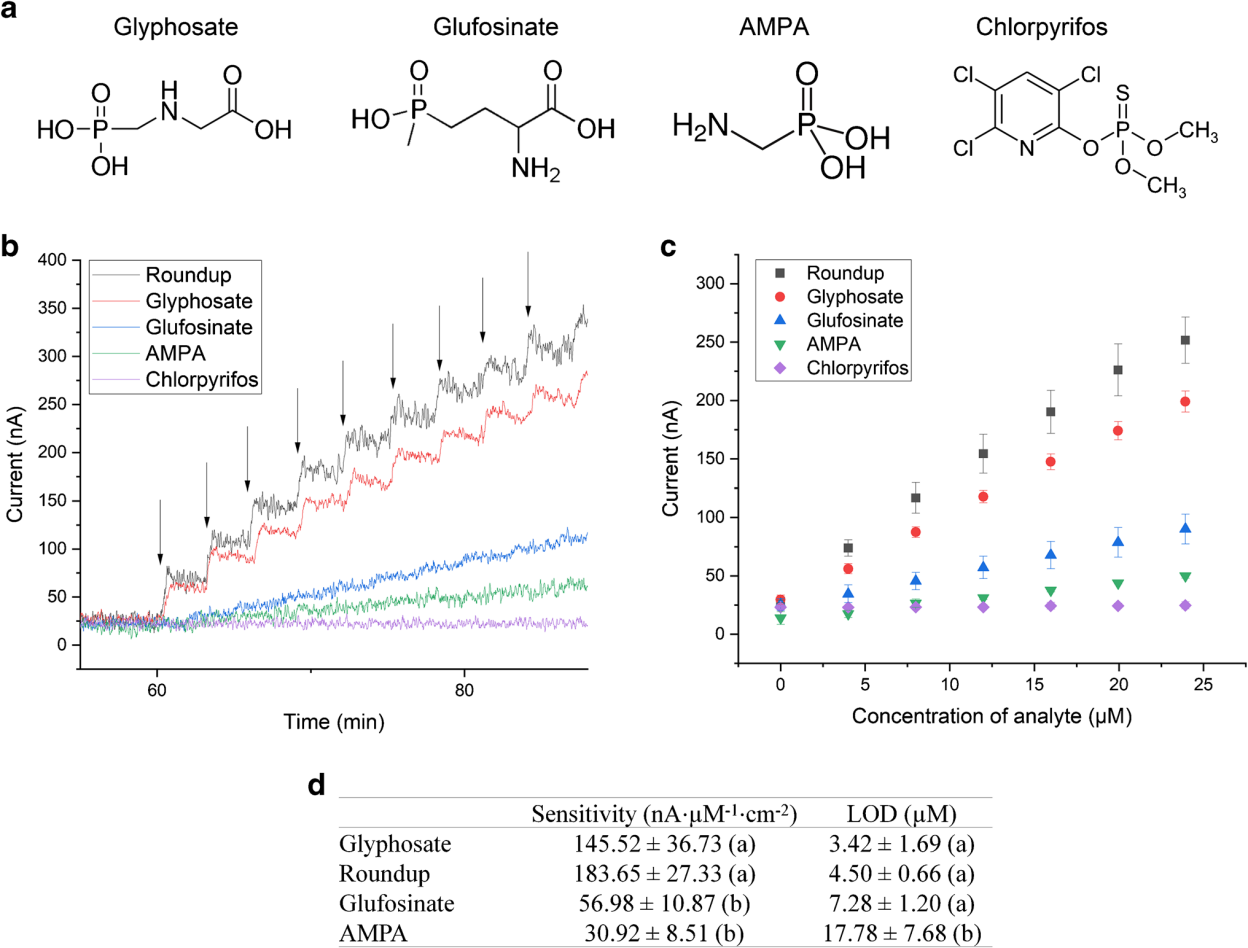
The **a** Chemical structure of tested organophosphorus compounds: glyphosate, glufosinate, aminomethylphosphonic acid (AMPA), and chlorpyrifos. **b** Representative amperometric response of LIG-Cu electrodes in PBS (pH 7.2) at a polarization potential of 100 mV (rolling average, *n* = 5). Black arrows represent injections of each one of the organophosphorus compounds to the electrochemical cell. **c** Calibration curve of LIG-Cu electrodes in the presence of different organophosphorus compounds. Error bars represent standard error (*n* ≥ 3) **d** Performance parameters of the sensor. Same letters represent groups with no significant difference for each variable

The LIG-Cu sensor was also tested for the detection of Roundup, which is a commercial glyphosate-based formulation used worldwide. Additionally, we evaluated the response to other organophosphorus compounds commonly found in agriculture-impacted waters where synthetic pesticides are routinely applied: AMPA, glufosinate-ammonium, and chlorpyrifos (Fig. 3a). Figure 3b depicts the real-time DCPA response of the LIG-Cu sensor exposed to increasing concentrations of different organophosphorus compounds. As seen in the figure, the current of the sensor did not change when chlorpyrifos was added to the electrochemical cell (purple curve in Fig. 3b), and the addition of AMPA and glufosinate-ammonium generated a much lower current response compared to Roundup and glyphosate. The performance parameters of the sensor for the organophosphorus compounds are summarized in Fig. 3d. The statistical analysis indicates that there is no significant difference between glyphosate and Roundup for the LOD and sensitivity (*p*⪫0.05), suggesting low interference from complex mixtures (e.g., the inactive ingredients in Roundup). Moreover, the sensitivity for AMPA and glufosinate-ammonium is significantly lower than the sensitivity with glyphosate; and the LOD for AMPA is significantly higher compared to glyphosate. In general, these results show a higher sensitivity of the sensor towards glyphosate than other organophosphorus compounds potentially present in real water samples. Our hypothesis is that phosphonic acid and carboxylic acid groups of glyphosate bind strongly to the copper oxide nanocrystals on the surface of the sensor. On the other hand, AMPA only has a phosphonic acid group, and glufosinate only has a carboxylic acid group, thus exhibiting a weaker binding with the sensor surface. Finally, the absence of acidic groups in chlorpyrifos explains the null current response from the sensor. Thus, demonstrating promising applicability of the sensor for testing environmental water without the need for sample pre-treatments such as filtration.

### Effect of different dissolved ions

**Fig. 4:**
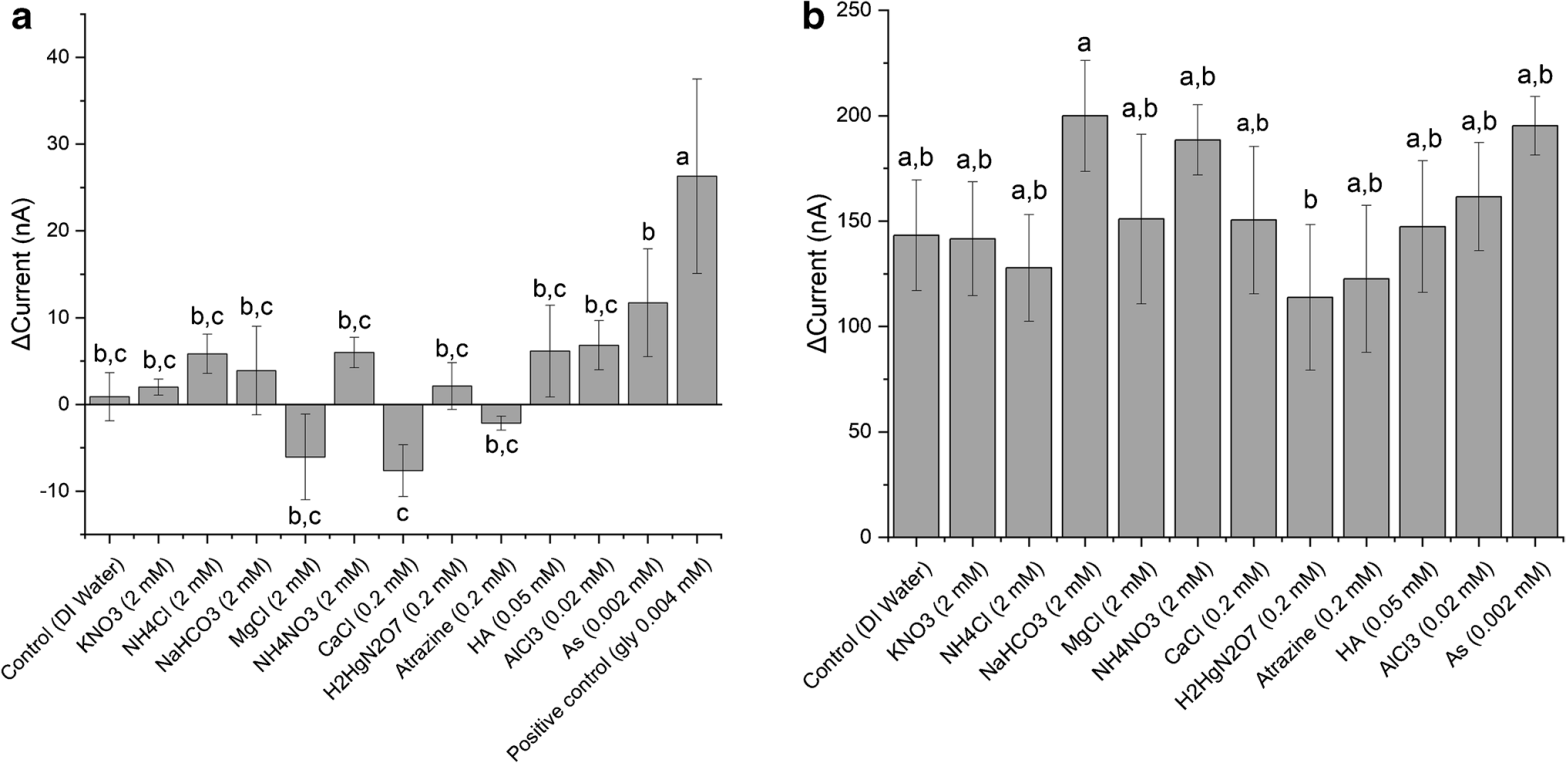
Matrix effects: **a** Difference in baseline current of the LIG-Cu sensor after the injection of possible interfering ions into the electrochemical cell filled with PBS buffer (pH 7.2) at a polarization potential of 100 mV. **b** Sensor response to glyphosate (final concentration of 19.9 µM) in the presence of possible interferents. Same letters represent groups with no significant difference (*p* < 0.05)

In the selectivity analysis, the change in current was reported in comparison to both negative and positive controls: DI water (Millipore, St. Louis, MO, USA) was used as negative control to determine whether dissolved ions generated a response significantly different than the normal background noise produced by the injection of DI water (Fig. 4a). The response of the sensor is not significantly affected by the presence of the tested ions at relevant concentrations in the environment. Concentrations tested are higher than concentrations typically found in environmental water samples, except for humic acid and calcium chloride, which were tested within the range of concentrations in the environment. The change in current is significantly lower than a positive control at low concentrations of glyphosate (4 μM), which is compatible with the MRL established by the EPA. Figure 4b shows that the capacity of the sensor to detect glyphosate at a higher concentration (20 μM) was not affected by the presence of the tested compounds.

It is worth noticing that the amperometric test was performed at a polarization potential of 100 mV. A low amperometric potential, as used in this and other studies [24–26], is advantageous to avoid oxidation of electroactive molecules potentially present in the water sample. For instance, ascorbic acid and uric acid can be oxidized at a minimum potential of + 400 mV in amperometric tests [27, 28].

### Stability and shelf life of LIG-Cu sensors

**Fig. 5:**
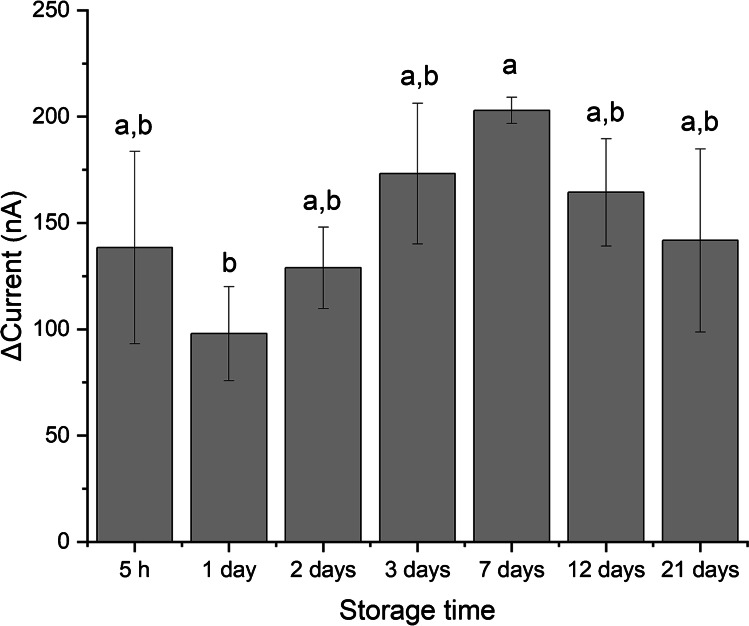
Difference in the amperometric current of the sensor in PBS solution at a polarization potential of 100 mV after the injection of glyphosate (final concentration of 19.9 µM) at different storage times. Same letters represent groups with no significant difference

To determine the extent to which the LIG-Cu sensors can be stored at ambient conditions after fabrication, the current response to glyphosate exposure was evaluated for sensors stored for different time spans up to 21 days. Upon fabrication, the electrodes were placed in a plastic Petri dish and stored in a cabinet in the laboratory. The air in the laboratory is maintained at a constant temperature of 23℃. The statistical analysis indicates that there are no significant differences in the response of the sensors stored between 5 h and 21 days after fabrication (Fig. 5). Future work must focus on enhancing the reproducibility of the manufacturing process in order to minimize the variability among LIG-Cu sensors. The observed variability in the current produced by the sensors may be explained by the lack of tight controls in environmental conditions, particularly relative humidity which has been shown to affect the behavior of graphene-based materials [29]. This issue may be resolved by storing the sensors in vacuum-sealed packages.

### Analytical application

To test the capacity of the sensor to detect glyphosate in environmental water samples, a calibration curve was obtained using glyphosate in simulated fresh water. The performance parameters of the sensor were not affected when glyphosate was diluted in PBS buffer or in synthetic fresh water (Fig. S8). Additionally, the sensor was challenged using a certified reference material (44,690-U 1000 μg⋅mL^−1^ glyphosate solution in distilled water) at different concentrations of glyphosate: 4, 12, and 20 µM. Recovery rates from 98.6 to 124.8% were obtained (Table 1), confirming the capacity of the sensor to detect glyphosate.

**Table 1:** Glyphosate concentration using a certified reference material. *RSD* recovery standard deviation

Concentration in the sample (µM)	Concentration found (µM)	Recovery (%)	RSD (%)
4	4.9	123.4	37.8
12	15.0	124.8	16.0
20	19.7	98.6	5.5

### Comparison with other sensors

Finally, while the LOD of the LIG-Cu sensor is higher than other sensors reported in the literature (Table 2), it is still useful for detecting glyphosate concentrations in water at the regulatory threshold established by the EPA. Even though some sensors use enzymes to obtain a sensible and selective analysis for glyphosate quantification [26], enzymatic sensors are susceptible to temperature damage, and require special storage conditions. Also, the potential presence of inhibitory molecules in the sample (e.g., heavy metals) may compromise the detection mechanisms [30]. Finally, the sensor described herein was fabricated with affordable materials (fabrication cost was estimated at $1.9 per sensor) and relatively simple fabrication methods. Thus, facilitating adoption and adaptation of the technology in regions where glyphosate testing is needed, but the manufacturing of exotic nanosensors may be completely unrealistic and perhaps unnecessary.

**Table 2:** Performance parameters of sensors for the detection of glyphosate. *NR* not reported

Type of sensor	Working electrode	LOD (µM)	Selectivity	Proof of concept	Reference
Electrochemical, amperometry	Rotating gold disk electrode modified with poly(2,5-dimethoxyaniline)-poly(4-styrenesulfonic acid) (PDMA-PSS) nanoparticles and immobilization of horseradish peroxidase (HRP)	0.0001	Glufosinate	Spiked corn samples from 2.0 to 78.0 µg L^−1^	[26]
Electrochemical, amperometry	Gold electrode with porous copper nanowire electrodeposition	0.01	NR	Fresh fruit and vegetable samples from non-detectable to (0.104 ± 0.005) µM	[14]
Electrochemical, LSV	Molecularly imprinted polymer-based electrochemical sensor	4.73 × 10^−9^	AMPA	Spiked tap water	[9]
Electrochemical, DPSV	Hierarchically porous Cu-BTC metal–organic framework platform	1.40 × 10^−7^	AMPA, pesticides and metal ions*	Soybeans with recovery rates 98–105%	[13]
Optic	Nanofiber sensor strips based on an optical color change of poly (vinyl) alcohol (cd-PVA (copper doped poly (vinyl) alcohol))	0.59	AMPA and glycine. Not selective (relative errors > 5%) in the presence of ions	Environmental water samples, with a recovery rate of 128.2% ± 3.1	[31]
SPR	Gold sensor chip decorated with an oligopeptide (TPFDLRPSSDTR)	0.58	NR	NR	[32]
Electrochemical, DPV	Glassy carbon electrode decorated with aluminum and copper nanocomposite with pristine graphene	0.0001	Glufosinate affected the response of the sensor. Other compounds† caused little effect on the response	Recovery rates in spiked surface water: 97.64 to 108.08%	[30]
Electrochemical, DPV	Glassy carbon modified with reduced graphene oxide and copper nanoparticles	0.19	Pesticides‡	Tap water. Recovery rate 96 and 104%	[33]
Electrochemical, DPV	Pencil graphite electrode coated by hollow fiber (HF) modified with copper oxide nanoparticles and multi-walled carbon nanotube (MWCNTS)-ionic liquid (IL)	0.0013	Inorganic ions and organic compounds§ produced an error of ≤ 5%	Spiked soil and river water samples with recovery rates between 92 and 103%	[34]
Electrochemical, amperometry	LIG decorated with copper nanoparticles	3.50 ± 1.70	Other pesticides and salts	Synthetic fresh water and certified reference material with recovery rates between 98.6 and 124.8%	This work

^*^Pesticides (trichlorfon, carbendazim, acetochlor and thiram) and metal ions (including K^+^, Ca^2+^, Zn^2+^, NO_3_^−^, Cl^−^, and SO_4_^2−^).

^†^Organophosphorus compounds and inorganic ions (carbonate, sulfate, nitrate, chloride, potassium, calcium, and sodium).

^‡^Pesticides (cypermethrin, deltamethrin, diazinon, malathion, mevinphos).

^§^Inorganic ions and organic compounds (glufosinate, bialapho, tridemorph, chlorpyrifos, cypermethrin, AMPA, Zn^2+^, Cd^2+^, Ca^2+^, Mg^2+^, Na^+^, NH^4+^, Br^−^, NO3^−^, SO_4_^2−^, PO_4_^3−^).

^‖^Pesticides: glufosinate, chlorpyrifos, AMPA, atrazine. Inorganic salts: potassium nitrate (KNO_3_), sodium bicarbonate (NaHCO_3_), calcium chloride (CaCl_2_), ammonium chloride (NH_4_Cl), magnesium chloride (MgCl_2_), aluminum chloride (AlCl_3_), arsenic, mercury (II) nitrate hydrate (H2HgN2O7).

*LSV* linear sweep voltammetry, *DPSV* differential pulse stripping voltammetry, *SPR* surface plasmon resonance, *DPV* differential pulse voltammetry.

## Conclusions

An electrochemical sensor was developed for the detection of organophosphorus pesticides with higher analytical sensitivity toward glyphosate. The sensing platform consists of laser-inscribed graphene electrodes decorated with copper nanoparticles. An amperometric test in PBS (pH 7.2) at a potential of +100 mV was used as the detection technique. The exposure of the electrodes to glyphosate results in an increase of the anodic current. When hooked to an affordable portable potentiostat, the LOD of the sensor is slightly lower than the MRL established by the EPA. The response of the sensor is pH dependent, and its highest analytical sensitivity was achieved when the working solution is maintained within the 7–8 pH range at standard temperature and pressure conditions. Future work will focus on sensor improvement in terms of reproducibility and stability. While the performance metrics of the sensor are not as impressive as other glyphosate sensors previously published, major advantages of the nanosensor presented herein are the following: (i) relatively facile manufacturing process, (ii) operational versatility to be used both in-laboratory and in-field settings, and (iii) overall lower cost compared with standard analytical techniques for pesticide testing. Thus, the developed sensor has the potential to be used for pollution monitoring in regions where heavy application of organophosphate pesticides may be of concern. In particular, for rural and low-income communities where glyphosate pollution represents an environmental and sociocultural problem.

## Supplementary Information

Below is the link to the electronic supplementary material.

Supplementary file1 (DOCX 1022 kb)

## References

[1] Benbrook CM (2016). Trends in glyphosate herbicide use in the United States and globally. Environ Sci Eur.

[2] World Health Organization (WHO)/International Agency for Research on Cancer (IARC) (2017) IARC Monographs on the evaluation of carcinogenic risks to humans. Volume 112. Some organophosphate insecticides and herbicides.

[3] Bai SH, Ogbourne SM (2016). Glyphosate: environmental contamination, toxicity and potential risks to human health via food contamination. Environ Sci Pollut Res.

[4] Acquavella JF, Alexander BH, Mandel JS, Gustin C, Baker B, Chapman P, Bleeke M (2004). Glyphosate biomonitoring for farmers and their families: results from the Farm Family Exposure Study. Environ Health Perspect.

[5] World Health Organization (WHO) 2015 IARC monographs Volume 112: evaluation of five organophosphate insecticides and herbicides Lyon France

[6] United States Environmental Protection Agency (EPA) (2018). 2018 Edition of the Drinking Water Standards and Health Advisories Tables.

[7] Council of the European Union (1998) COUNCIL DIRECTIVE 98/83/EC of 3 November 1998 on the quality of water intended for human consumption. Official Journal of the European Communities. Council of the European Union

[8] United States Environmental Protection Agency (EPA) (1993) Glyphosate. R.E.D. FACTS. Prevention, Pesticides And Toxic Substances (7508W). EPA-738-F-93–011 September 1993

[9] Do MH, Florea A, Farre C, Bonhomme A, Bessueille F, Vocanson F, Tran-Thi N-T, Jaffrezic-Renault N (2015). Molecularly imprinted polymer-based electrochemical sensor for the sensitive detection of glyphosate herbicide. Int J Environ Anal Chem.

[10] Sakamoto S, Putalun W, Vimolmangkang S, Phoolcharoen W, Shoyama Y, Tanaka H, Morimoto S (2018). Enzyme-linked immunosorbent assay for the quantitative/qualitative analysis of plant secondary metabolites. J Nat Med.

[11] Ronkainen NJ, Halsall HB, Heineman WR (2010). Electrochemical biosensors. Chem Soc Rev.

[12] Moraes FC, Mascaro LH, Machado SA, Brett CM (2010). Direct electrochemical determination of glyphosate at copper phthalocyanine/multiwalled carbon nanotube film electrodes. Electroanalysis.

[13] Cao Y, Wang L, Wang C, Hu X, Liu Y, Wang G (2019). Sensitive detection of glyphosate based on a Cu-BTC MOF/g-C3N4 nanosheet photoelectrochemical sensor. Electrochim Acta.

[14] Poorahong S, Thammakhet C, Thavarungkul P, Kanatharana P (2015). One-step preparation of porous copper nanowires electrode for highly sensitive and stable amperometric detection of glyphosate. Chem Pap.

[15] Hurtado-Bermúdez LJ, Vélez-Torres I, Méndez F (2020). No land for food: prevalence of food insecurity in ethnic communities enclosed by sugarcane monocrop in Colombia. Int J Public Health.

[16] Sheals J, Persson P, Hedman B (2001). IR and EXAFS spectroscopic studies of glyphosate protonation and copper (II) complexes of glyphosate in aqueous solution. Inorg Chem.

[17] Tehrani F, Bavarian B (2016). Facile and scalable disposable sensor based on laser engraved graphene for electrochemical detection of glucose. Sci Rep.

[18] Vanegas D, Patiño L, Mendez C, Oliveira D, Torres A, Gomes C, McLamore E (2018). Laser scribed graphene biosensor for detection of biogenic amines in food samples using locally sourced materials. Biosensors.

[19] Jenkins DM, Lee BE, Jun S, Reyes-De-Corcuera J, McLamore ES (2019). ABE-Stat, a fully open-source and versatile wireless potentiostat project including electrochemical impedance spectroscopy. J Electrochem Soc.

[20] Mogera U, Dhanya R, Pujar R, Narayana C, Kulkarni GU (2015). Highly decoupled graphene multilayers: turbostraticity at its best. J Phys Chem Lett.

[21] Athanasiou M, Samartzis N, Sygellou L, Dracopoulos V, Ioannides T, Yannopoulos SN (2021). High-quality laser-assisted biomass-based turbostratic graphene for high-performance supercapacitors. Carbon.

[22] Huang L, Lee E-S, Kim K-B (2005). Electrodeposition of monodisperse copper nanoparticles on highly oriented pyrolytic graphite electrode with modulation potential method. Colloids Surf A Physicochem Eng Aspects.

[23] Beverskog B, Puigdomenech I (1997). Revised Pourbaix diagrams for copper at 25 to 300 C. J Electrochem Soc.

[24] Songa EA, Arotiba OA, Owino JH, Jahed N, Baker PG, Iwuoha EI (2009). Electrochemical detection of glyphosate herbicide using horseradish peroxidase immobilized on sulfonated polymer matrix. Bioelectrochemistry.

[25] Khenifi A, Derriche Z, Forano C, Prevot V, Mousty C, Scavetta E, Ballarin B, Guadagnini L, Tonelli D (2009). Glyphosate and glufosinate detection at electrogenerated NiAl-LDH thin films. Anal Chim Acta.

[26] Sok V, Fragoso A (2019). Amperometric biosensor for glyphosate based on the inhibition of tyrosinase conjugated to carbon nano-onions in a chitosan matrix on a screen-printed electrode. Microchim Acta.

[27] Yang M, Yang Y, Liu B, Shen G, Yu R (2004). Amperometric glucose biosensor based on chitosan with improved selectivity and stability. Sens Actuators B Chem.

[28] Yuan CJ, Hsu CL, Wang SC, Chang KS (2005). Eliminating the interference of ascorbic acid and uric acid to the amperometric glucose biosensor by cation exchangers membrane and size exclusion membrane. Electroanalysis: Int J Devoted Fundam Pract Aspects Electroanal.

[29] Qadir A, Sun Y, Liu W, Oppenheimer PG, Xu Y, Humphreys C, Dunstan D (2019). Effect of humidity on the interlayer interaction of bilayer graphene. Phys Rev B.

[30] Zhang C, Liang X, Lu Y, Li H, Xu X (2020). Performance of CuAl-LDH/Gr Nanocomposite-based electrochemical sensor with regard to trace glyphosate detection in water. Sensors.

[31] De Almeida L, Chigome S, Torto N, Frost C, Pletschke B (2015). A novel colorimetric sensor strip for the detection of glyphosate in water. Sensors Actuators B Chem.

[32] Ding X, Yang K-L (2013). Development of an oligopeptide functionalized surface plasmon resonance biosensor for online detection of glyphosate. Anal Chem.

[33] Setznagl S, Cesarino I (2020). Copper nanoparticles and reduced graphene oxide modified a glassy carbon electrode for the determination of glyphosate in water samples. Int J Environ Anal Chem.

[34] Gholivand M-B, Akbari A, Norouzi L (2018). Development of a novel hollow fiber-pencil graphite modified electrochemical sensor for the ultra-trace analysis of glyphosate. Sens Actuators B Chem.

