# Supplementary material for: Development of a nanocopper-decorated laser-scribed sensor for organophosphorus pesticide monitoring in aqueous samples
Source: Mikrochim Acta. 2022 Jun 13;189(7):254. doi: 10.1007/s00604-022-05355-w (PMC9192389; doi:10.1007/s00604-022-05355-w)
Supplement: Supplementary file 1 — Supplementary file1 (DOCX 1022 kb) [file 604_2022_5355_MOESM1_ESM.docx]

**Electronic Supplementary Material**

**Development of a nanocopper-decorated laser-scribed sensor for organophosphorus pesticide monitoring in aqueous samples**

D. Bahamon-Pinzon (ORCID: 0000-0003-2149-1964)^1,4^; Geisianny Moreira (ORCID: 0000-0002-7008-0363)^1,4^; Sherine Obare (ORCID: 0000-0003-3317-7147)^2^; D.C. Vanegas (ORCID 0000-0001-9858-0960)^1,3,4*^

^1^ Department of Environmental Engineering and Earth Sciences, Clemson University, SC, USA.

^2^ Joint School of Nanoscience and Nanoengineering; North Carolina A&T State University and UNC Greensboro, NC, USA.

^3^ Interdisciplinary Group for Biotechnology Innovation and Ecosocial Change -BioNovo, Universidad del Valle, Cali, Colombia.

^4^ Global Alliance for Rapid Diagnostics, Michigan State University, USA

*Corresponding author: Diana Vanegas dvanega@clemson.edu

**Methodology**

**Preparation of sensing platform**


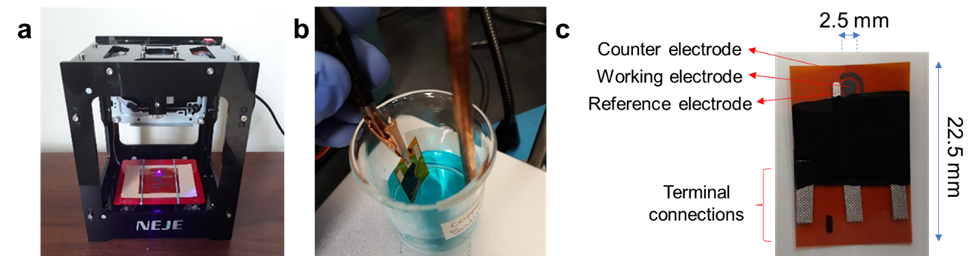


**Fig. S1** Fabrication process of the sensing platform. **a** Fabrication of turbostratic graphene electrodes via laser inscribing, **b** copper electrodeposition of the working electrode in a solution of 250 mM copper sulfate (CuSO_4_) and 2.5 mM sodium sulfate (Na_2_SO_4_) using a copper rod and **c** three-electrode system

**Quality control process and selection of replicates**

All fabricated sensors were tested via cyclic voltammetry in PBS using a portable potentiostat (ABE-Stat, MultiPalmSens4, PalmSens, Houten, Netherlands) with a potential range of 0 to 0.5 V, scan rate of 200 mV⋅s^-1^, and 10 cycles. This process was applied to clean the electrode surface since the repetitive potential sweeps help remove any loosely attached particles from the electrode's surface. Electrodes were selected based on a qualitative analysis of the level of overlap of the last (i.e., the 10th) cyclic voltammograms (Fig. S2). The selection was verified by confirming that the coefficient of variation (standard deviation x 100/average) of the oxidation current at 0.25 V was lower than 80%.


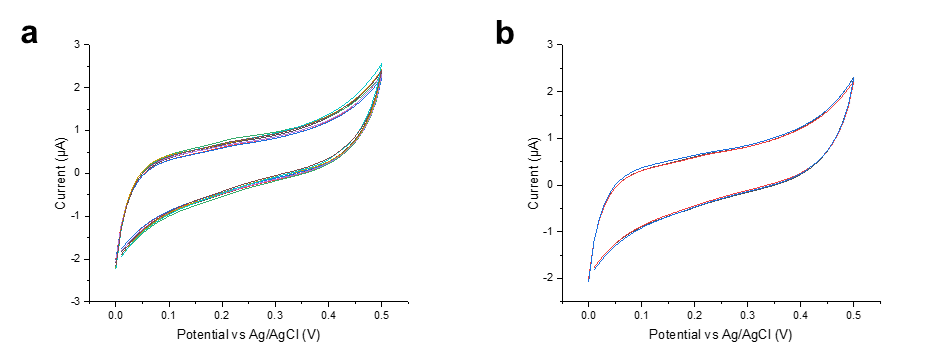


**Fig. S2** Representative selection of electrodes with similar electrochemical response. **a** Last cyclic voltammogram of 10 cycles for eight electrodes in PBS solution with a potential range of 0 to 0.5 V at 200 mV⋅s^-1^. **b** Cyclic voltammogram of three electrodes selected for analysis.

**Electrochemical characterization: Cyclic Voltammetry**

Cyclic voltammetry was performed using a benchtop potentiostat (MultiPalmSens4, PalmSens, Houten, Netherlands) coupled to an electrochemical cell Stand (EF-1085 C-3, BASi, EF-1085 C-3, BASi West Lafayette, IN, USA). Cyclic voltammetry was used to determine the electroactive surface area of the working electrode, using a potential range from -0.4 V to 0.8 V at different scan rates (20, 50, 100, 125, 150, and 200 mV⋅s^-1^). During the test, the sensors were immersed in a 4 mM potassium ferrocyanide (K_4_Fe(CN)_6_) and 1 M potassium nitrate (KNO_3_) solution. The electroactive area of the sensors was determined through the Randles-Sevcik model (Eq. S1)^[[1]](#footnote-1)^, using the slope of the Cottrell plot (linear correlation between the peak of the current, i_p_, and the square root of the scan rate, v^1/2^). The number of electrons in the redox reaction, n, the diffusion coefficient, D, and the concentration, C, are constants (n=1, D=6.7 cm^2^⋅s^-1^, C=4x10^-6^ mol⋅cm^-3^).

$$ESA=i_{p}/(2.69x{10}^{5})n^{3/2}D^{1/2}C v^{1/2} (Eq.S1)$$

Where:

ESA: electroactive surface area (cm^2^).

i_p_: peak current (A).

n: number of electrons in the redox reaction.

v: scan rate (V⋅s^-1^).

D: diffusion coefficient (cm^2^⋅s^-1^).

C: Concentration (mol⋅cm^-3^).

**Effect of pH in electrochemical performance of LIG-Cu electrodes**

The effect of the solution pH on the electrochemical response of the LIG-Cu electrode was investigated using PBS buffer (pH 7.2) and sodium hydroxide and hydrochloric acid to adjust the pH of the supporting electrolyte to 5, 6, and 8 (this pH range is relevant to environmental water samples). The current of the sensor was recorded during an amperometric test involving a single 50 µL addition of glyphosate (8 µM) into the working solution (20 mL PBS) to obtain a final concentration of glyphosate in the electrochemical cell of 19.9 µM, which is within the linear range of the calibration curve of the sensor.

## **Selectivity and stability analysis**

Amperometry was used to test the selectivity of the sensor towards glyphosate. After 60 min of polarization, an aliquot of the interferent was added. Distilled water was used as control. Three minutes later, glyphosate was injected into the electrochemical cell. The sensor response was recorded in the presence of the following compounds: potassium nitrate (KNO_3_), sodium bicarbonate (NaHCO₃), calcium chloride (CaCl_2_), ammonium chloride (NH_4_Cl), magnesium chloride (MgCl_2_), aluminum chloride (AlCl_3_), arsenic, mercury (II) nitrate hydrate (H_2_HgN_2_O_7_), and atrazine. For stability analysis, sensors were stored at room temperature and tested on the same day as fabricated and 1, 2, 3, 7, and 21 days after fabrication in terms of current after the addition of an aliquot of glyphosate during an amperometric test. Different batches of three electrodes were prepared for each day of analysis.

**Protocol for analysis of water samples**

The sensor was tested with synthetic fresh water (1.14 mM sodium bicarbonate, 0.44 mM calcium sulfate, 0.50 mM magnesium sulfate, and 0.05 mM potassium chloride^[[2]](#footnote-2)^). The LOD and sensitivity were determined using PBS as supporting electrolyte, and a glyphosate salt dissolved in synthetic fresh water was used as a stock solution (8 mM) to simulate the conditions of herbicide-contaminated water. The sensor was also tested using a certified reference material for glyphosate. After 60 minutes of polarization using PBS as supporting electrolyte, different volumes were added to the electrochemical cell to obtain concentrations of 4 μM, 12 μM and 20 μM of glyphosate.

For analysis of an unknown sample, the first step is to polarize the electrodes system prior to the amperometric test by immersing the electrodes in a volume (V_t_) of 20 mL PBS buffer (pH 7.2). Counter, reference and working electrodes must be properly connected to the potentiostat, and a constant potential of +100 mV must be applied throughout the entire duration of the test. The electrodes are sufficiently polarized after 60 minutes. This can be visualized on the signal acquisition system as a flat current baseline (less than 5% drift in current). The next step is to inject a specific volume of sample (V_s_) (not exceeding 400 $\mu$L). Allow three minutes for the current response to stabilize (approx. 3 min). The concentration of the organophosphorus compound in the electrochemical cell (C_t_) can be calculated based on the stable current response obtained, which is then contrasted with the calibration curve (Fig. 2b). Finally, the sample concentration (C_s_) can be calculated according to the following expression:

$$C_{s} \left[ \mu M \right]= \frac{C_{t} \left[ \mu M \right] V_{t} [mL]}{V_{s} [mL]}$$

**Statistical analysis**

**Table S1** Parameters used for statistical analyses

| Statistical test | Factor | Levels | Response variables |
| --- | --- | --- | --- |
| ANOVA/Tukey | Analyte | Roundup, glyphosate, glufosinate-ammonium, and AMPA | 1) Sensitivity and 2) LOD |
| ANOVA/Tukey | pH of supporting electrolyte | 5, 6, 7, and 8 | Change in current after addition of glyphosate |
| ANOVA/Tukey | Possible interfering compounds | Control (distilled water), potassium nitrate (KNO_3_), sodium bicarbonate (NaHCO₃), calcium chloride (CaCl_2_), ammonium chloride (NH_4_Cl), magnesium chloride (MgCl_2_), aluminum chloride (AlCl_3_), arsenic, mercury (II) nitrate hydrate (H_2_HgN_2_O_7_), ammonium nitrate (NH_4_NO_3_), atrazine, and humic acid. | 1) Change in current after the addition of interfering compound and 2) change in current after addition of glyphosate |
| ANOVA/Tukey | Storage time | 5 h, 1 day, 2 days, 3 days, 7 days, 12 days, and 21 days | Change in current after addition of glyphosate |
| t test | Matrix to prepare glyphosate solution | PBS and synthetic fresh water | 1) Sensitivity and 2) LOD |

**Results**

**Materials characterization**


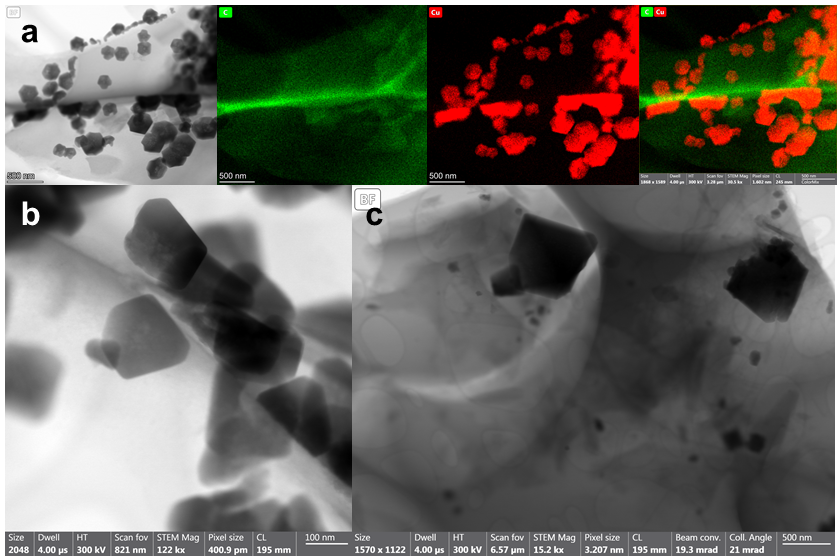


**Fig. S3 a** Composition maps of carbon in green (C) and copper in red (Cu) in the surface of LIG-Cu electrodes obtained with electron diffraction spectroscopy (EDS). Scanning Transmission Electron Microscopy (STEM) images of LIG-Cu electrodes at a magnification of **b** 122 kx and **c** 15 kx


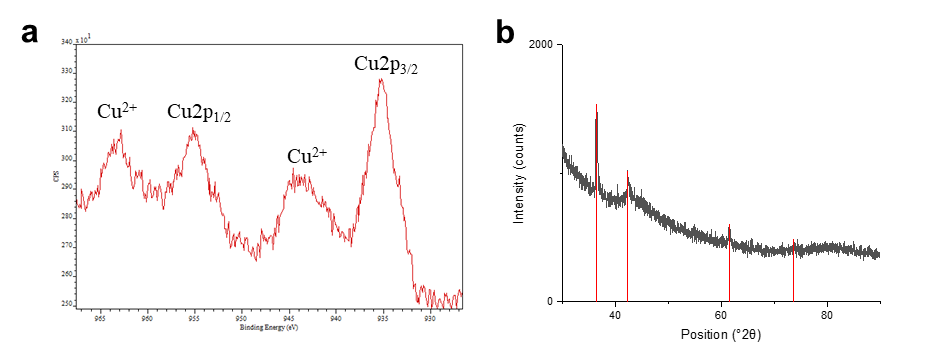


**Fig. S4 a** X-ray photoelectron spectroscopy (XPS) spectrum of LIG-Cu electrodes. **b** X-ray Diffraction of LIG-Cu electrodes. Red lines indicate peaks corresponding to the spectrum of copper oxide.

**Electroactive surface area**

Cyclic voltammograms of LIG electrodes show two well-defined oxidation and reduction peaks at 0.33 V and -0.02 V, respectively (Fig. S5c). While LIG-Cu electrodes also have two well-defined redox peaks at 0.49 V and -0.13 V that can be attributed to the ferrocyanide couple, an additional reduction peak appears at 0.45 V (Fig. S5b,c). Another oxidation peak for copper appears at 0.45 V in the cyclic voltammogram with a wider potential range (Fig. S5e). The redox peaks are associated with the presence of copper oxide, which can undergo electrochemical reduction within the potential range tested. Yadav and Lee (2019) studied the electrocatalytic activity of copper nanoparticles in glass substrates in 0.1 M sodium hydroxide using cyclic voltammetry and obtained three oxidation peaks that correspond to the formation of copper(I) oxide (Cu_2_O), a combination of copper(II) oxide and copper(II) hydroxide, and copper(III) oxide (CuOOH) and two reduction peaks that are due to the transformation of copper oxide and copper hydroxide to copper(II) oxide^[[3]](#footnote-3)^.

The current of the oxidation and reduction peaks associated with the ferrocyanide probe increased with the scan rate for both LIG and LIG-Cu (Fig. S5a and b). Cottrell plots show a linear relationship between the current of oxidation peaks and the square root of the scan rate (R^2^=0.9995 for both LIG and LIG-Cu, Fig. S5d). The slope of the Cottrell plots was used to calculate the Electroactive Surface Area (ESA). The ESA of LIG-Cu (0.035 ± 0.004 cm^2^) was higher than LIG (0.026 ± 0.002 cm^2^), which indicates a higher conductivity of the LIG-Cu electrodes. The heterogeneous electron transfer (HET) constant was not calculated using the Nicholson method^[[4]](#footnote-4)^ because the potential separation was higher than 300 mV (ΔE=346.67 ± 15.28 for LIG and ΔE=626.33 ± 166.85 for LIG-Cu), indicating slow electron transfer kinetics.


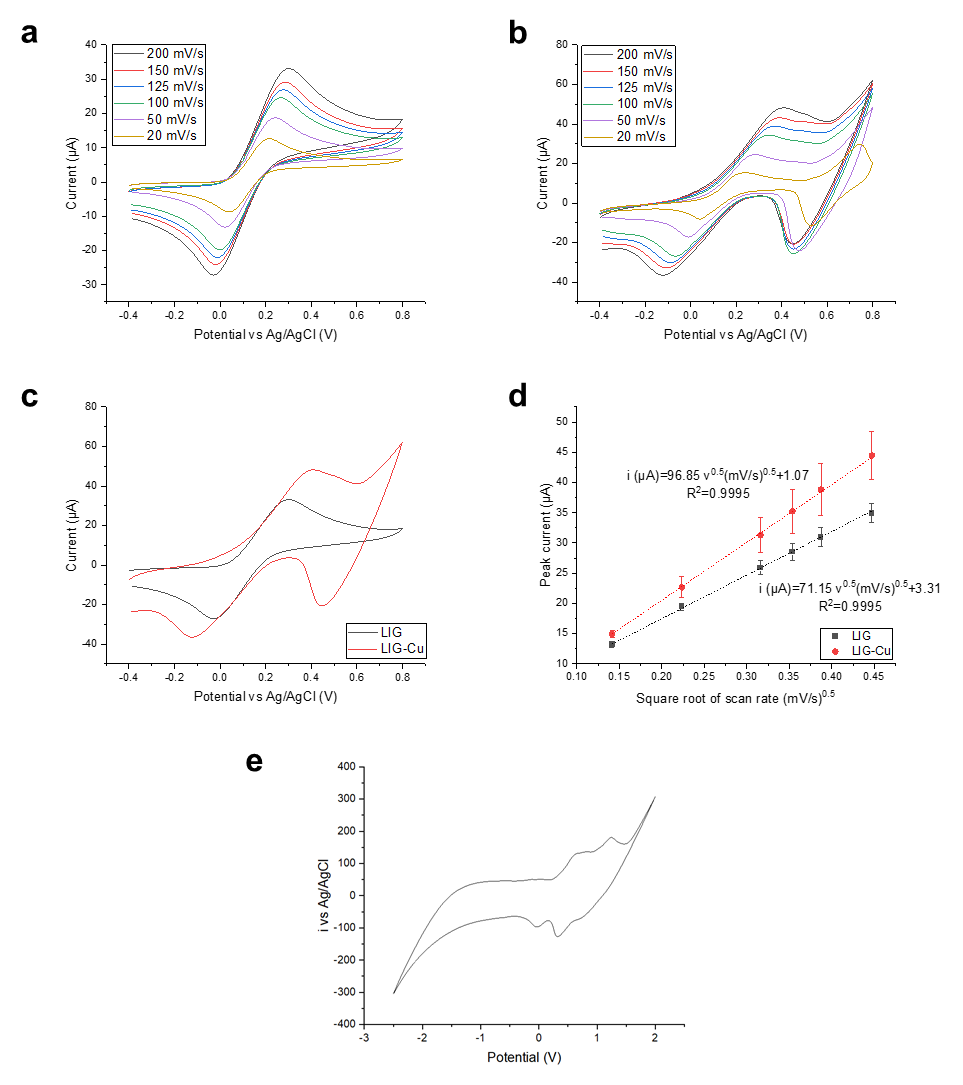


**Fig. S5** Representative cyclic voltammograms at different scan rates of **a** laser inscribed graphene (LIG) and **b** LIG with copper nanoparticles (LIG-Cu) electrodes. **c** Comparison of cyclic voltammograms at 200 mV⋅s^-1^ of LIG and LIG-Cu. Cyclic voltammetry (a-c) was performed in a 4 mM potassium ferrocyanide (K_4_Fe(CN)_6_) and 1 M potassium nitrate (KNO_3_) solution. **d** Cottrell plots of LIG and LIG-Cu. **e** Representative cyclic voltammogram at 200 mV⋅s^-1^ of LIG-Cu electrodes in a 4 mM potassium ferrocyanide (K_4_Fe(CN)_6_) and 1 M potassium nitrate (KNO_3_) solution.


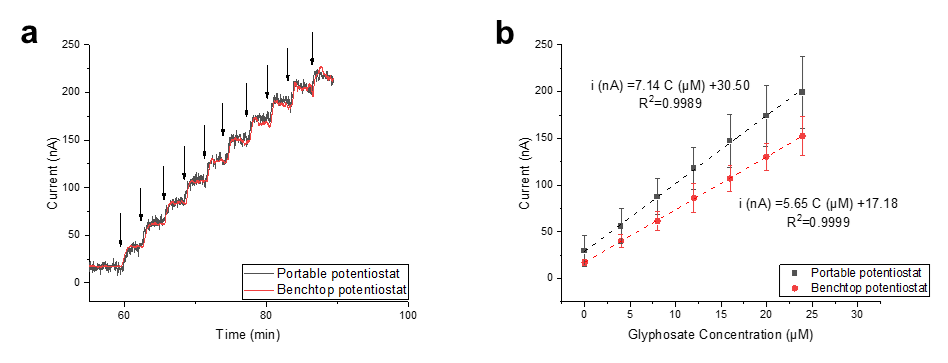


**Fig. S6** **a** Representative amperometric response of LIG-Cu electrodes in PBS (pH 7.2) at a polarization potential of 100 mV using a portable potentiostat (ABE-Stat, black curve) and a benchtop potentiostat (MultiPalmSens4, red curve) (rolling average, n=5). The black arrows represent injections of glyphosate diluted in PBS and in synthetic water to the electrochemical cell. **b** Calibration curve of the sensor in the presence of different organophosphorus compounds. Error bars represent standard error (n≥3).

Fig. S7 shows the DCPA response of the LIG-Cu electrode after injecting glyphosate into the working buffer solution with different pH values. As observed in the figure, the highest amperometric response from a single injection of analyte is obtained within the pH range from 7 to 8. At lower solution pH, the response of the sensor decreased significantly. This effect can be due to the change in surface charge of both the composite electrode and the glyphosate molecule. At low pH, glyphosate has less negatively charged functional groups, which may limit the interaction with positively charged compounds, such as copper. Therefore, we selected the pH 7 for the supporting electrolyte for future experiments. These results indicate the importance of working with a buffer to maintain the pH of the solution at a relatively stable pH level.


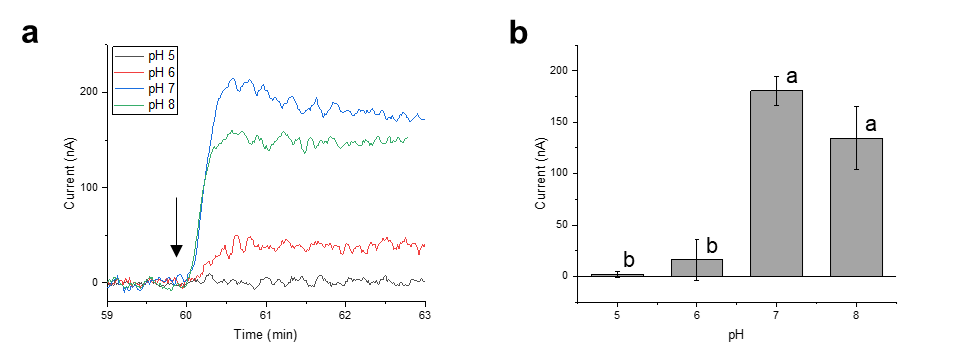


**Fig. S7 a** Representative amperometric response of LIG-Cu electrodes in PBS (pH 7.2) at a polarization potential of 100 mV (rolling average, n=5). The black arrow represents an injection of glyphosate to the electrochemical cell (final concentration of 19.9 µM). **b** Difference in the current of the sensor after the injection of glyphosate at different pH values. Same letters represent groups with no significant difference.


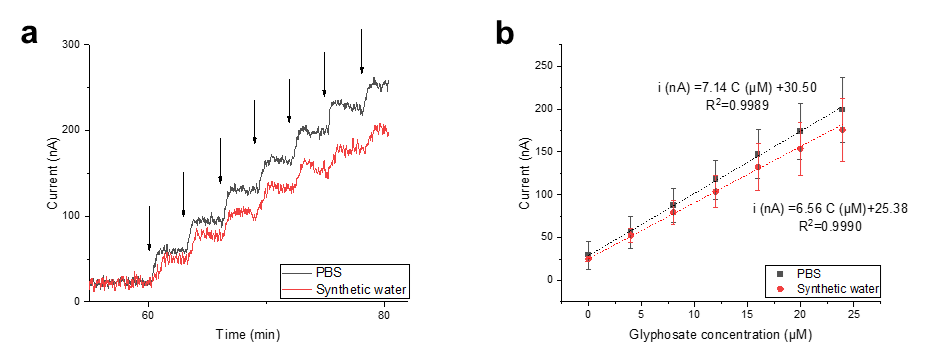


**Fig. S8** **a** Representative amperometric response of LIG-Cu electrodes in PBS (pH 7.2) at a polarization potential of 100 mV (rolling average, n=5). The black arrows represent injections of glyphosate diluted in PBS and in synthetic water to the electrochemical cell. **b** Calibration curve of the sensor when glyphosate is diluted in PBS or synthetic fresh water. Error bars represent standard error (n≥3).

1. Bard AJ, Faulkner LR (2000) Electrochemical Methods: Fundamentals and Applications. 2nd Edition. John Wiley & Sons, Incorporated [↑](#footnote-ref-1)
2. United States Environmental Protection Agency (EPA) (2002) Office of Water: Methods for Measuring the Acute Toxicity of Effluents and Receiving Waters to Freshwater and Marine Organisms. [↑](#footnote-ref-2)
3. Yadav HM, Lee J-J (2019) One-pot synthesis of copper nanoparticles on glass: applications for non-enzymatic glucose detection and catalytic reduction of 4-nitrophenol. Journal of Solid State Electrochemistry 23(2):503-512 [↑](#footnote-ref-3)
4. Nicholson RS (1965) Theory and application of cyclic voltammetry for measurement of electrode reaction kinetics. Analytical Chemistry 37(11):1351-1355 [↑](#footnote-ref-4)
